# 54770 A Comprehensive Online Platform for Plain Language Clinical Trial Result Summary Development

**DOI:** 10.1017/cts.2021.501

**Published:** 2021-03-30

**Authors:** Christian Reyes

**Affiliations:** University of Southern California

## Abstract

ABSTRACT IMPACT: This work will impact participants of clinical trials by better informing them of their trial’s results and their important role within the clinical research process. OBJECTIVES/GOALS: This project aims to equip researchers with an online tool for the development, dissemination, and collection of participant feedback for plain language clinical trial (CT) result summaries (PLCTRS). PLCTRS ensure that participants fully understand their trial’s results and their role in the CT process. METHODS/STUDY POPULATION: This online development platform is a web application made with CSS, HTML, and JavaScript. First, general trial identification information including study aims and eligibility will be input by researchers. Then, they will be prompted with tips and suggestions for composing in plain language and to ensure inclusion of all essential trial information. Next, the platform will disseminate this writing electronically to participants. Participants then provide meaningful feedback on the platform about their comprehension for the researcher, which the platform will aggregate and summarize for revisions. This process of drafting and feedback is repeated until a satisfactory PLCTRS is finalized. RESULTS/ANTICIPATED RESULTS: The anticipated results of this project are overall improved comprehension of clinical trial results by participants. This comprehension will be measured by participants ability to answer certain questions not only regarding trial outcomes, but also about the trial in general. For instance, before and after interacting with the PLCTRS, trial participants will be asked if they can identify which investigational drug was being studied and its possible side effects. This project could anticipate identification of trial elements and results deemed difficult to comprehend by participants; thus they would be better informed after interacting with the platform. DISCUSSION/SIGNIFICANCE OF FINDINGS: This project will ensure that participants better comprehend the trials they participated in beyond the required informed consent process - which only covers their comprehension prospectively. This project seeks to address the gap of ensuring participant comprehension retrospectively.

